# 1-(4-Chloro­phen­yl)-1*H*-1,2,4-triazol-5(4*H*)-one

**DOI:** 10.1107/S1600536814006412

**Published:** 2014-03-29

**Authors:** Pramod P. Kattimani, Ravindra R. Kamble, Mahadev N. Kumbar, H. K. Arunkashi, H. C. Devarajegowda

**Affiliations:** a Department of Studies in Chemistry, Karnataka University, Dharwad 580 003, Karnataka, India; b Department of Physics, Moodlakatte Institute of Technology, Kundapura 576 217, Karnataka, India; c Department of Physics, Yuvaraja’s College (Constituent College), University of Mysore, Mysore 570 005, Karnataka, India

## Abstract

In the title compound, C_8_H_6_ClN_3_O, the dihedral angle between the 1,2,4-triazole and benzene rings is 4.60 (9)° and an intra­molecular C—H⋯O inter­action closes an *S*(6) ring. In the crystal, inversion dimers linked by pairs of N—H⋯O hydrogen bonds generate *R*_2_^2^(8) loops and C—H⋯O inter­actions link the dimers into [100] chains. Weak π–π stacking inter­actions [centroid–centroid distance = 3.644 (1) Å] are also observed.

## Related literature   

For a related structure and background to 1,2,4-triazoles, see: Devarajegowda *et al.*(2012 ▶).
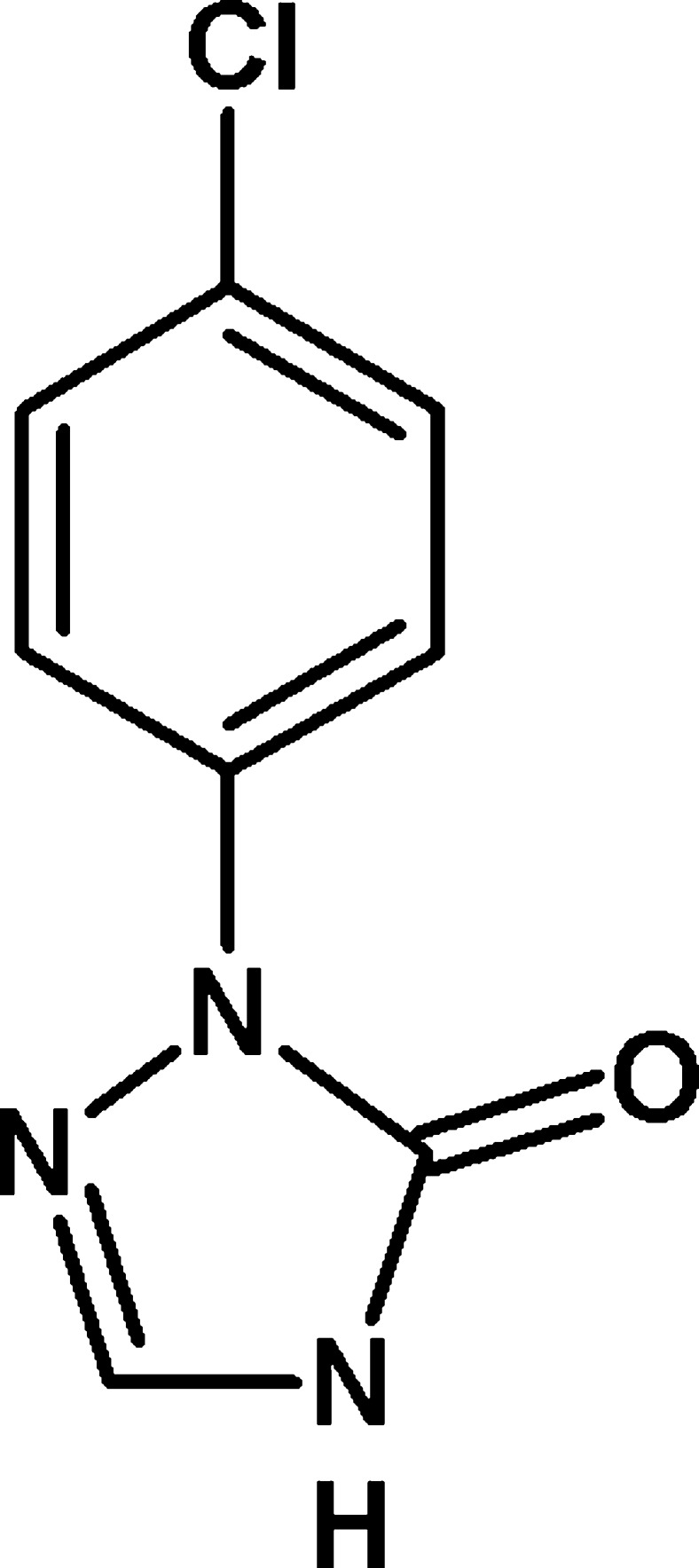


## Experimental   

### 

#### Crystal data  

C_8_H_6_ClN_3_O
*M* *_r_* = 195.61
Triclinic, 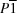

*a* = 6.5791 (4) Å
*b* = 7.2663 (4) Å
*c* = 9.3342 (5) Å
α = 80.121 (4)°
β = 85.042 (4)°
γ = 70.235 (4)°
*V* = 413.52 (4) Å^3^
*Z* = 2
Mo *K*α radiation
μ = 0.42 mm^−1^
*T* = 296 K
0.24 × 0.20 × 0.12 mm

#### Data collection  

Bruker SMART CCD diffractometer
Absorption correction: ψ scan (*SADABS*; Sheldrick, 2007 ▶) *T*_min_ = 0.770, *T*_max_ = 1.000
5938 measured reflections
1438 independent reflections
1270 reflections with *I* > 2σ(*I*)
*R*_int_ = 0.025

#### Refinement  

*R*[*F* ^2^ > 2σ(*F* ^2^)] = 0.034
*wR*(*F* ^2^) = 0.094
*S* = 1.06
1438 reflections
118 parameters
H-atom parameters constrained
Δρ_max_ = 0.17 e Å^−3^
Δρ_min_ = −0.15 e Å^−3^

### 

Data collection: *SMART* (Bruker, 2001 ▶); cell refinement: *SAINT* (Bruker, 2001 ▶); data reduction: *SAINT*; program(s) used to solve structure: *SHELXS97* (Sheldrick, 2008 ▶); program(s) used to refine structure: *SHELXL97* (Sheldrick, 2008 ▶); molecular graphics: *ORTEP-3 for Windows* (Farrugia, 2012 ▶); software used to prepare material for publication: *SHELXL97*.

## Supplementary Material

Crystal structure: contains datablock(s) I, global. DOI: 10.1107/S1600536814006412/hb7211sup1.cif

Structure factors: contains datablock(s) I. DOI: 10.1107/S1600536814006412/hb7211Isup2.hkl

Supporting information file. DOI: 10.1107/S1600536814006412/hb7211Isup3.cml

CCDC reference: 993126

Additional supporting information:  crystallographic information; 3D view; checkCIF report

## Figures and Tables

**Table 1:** Hydrogen-bond geometry (Å, °)

*D*—H⋯*A*	*D*—H	H⋯*A*	*D*⋯*A*	*D*—H⋯*A*
N5—H5⋯O2^i^	0.86	1.95	2.7924 (18)	166
C6—H6⋯O2^ii^	0.93	2.53	3.360 (3)	149
C9—H9⋯O2	0.93	2.29	2.933 (2)	126

Symmetry codes: (i) 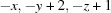; (ii) 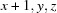.

**Fig. 1.**
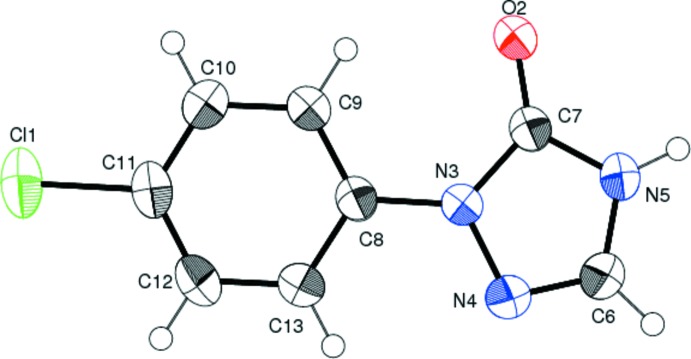
The molecular structure of the title compound. Displacement ellipsoids are drawn at the 50% probability level.

**Fig. 2.**
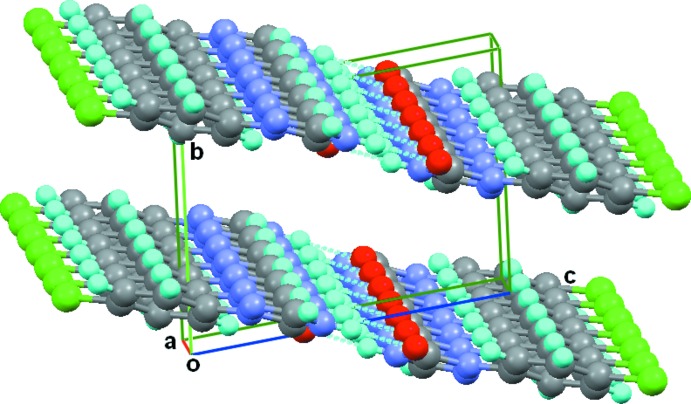
The packing of the title compound.

## supplementary crystallographic information

### 1. Comment

As part of our onging studies of 1,2,4-triazoles (Devarajegowda *et al.*, 2012), we now describe the synthesis and structure of the title compound.

The asymmetric unit of 2-(4-chlorophenyl)-2,4-dihydro-3*H*-1,2,4-triazol- 3-one is shown in Fig. 1. The dihedral angle between the 1,2,4-triazol ring (N3/N4/N5/C6/C7) and the benzene ring (C8–C13) is 4.60 (9)°. In the crystal, inversion related N5—H5···O2 interactions generate an *R*_2_^2^(8) ring pattern and link pairs of independent molecules into dimers and C6—H6···O interactions generate *R*_2_^2^(10) ring motifs. C_g_(1)π–πC_g_(2) interactions [centroid–centroid distance = 3.644 (1) Å] between 1,2,4 triazole (N3/N4/N5/C6/C7) and benzene (C8–C13) rings are also observed.

### 2. Experimental

2-(4-Chlorophenyl)-2,4-dihydro-3*H*-1,2,4-triazol-3-one was refluxed with formamide at 453 K. After completion of the reaction, the reaction mixture was poured into ice cold water to recover the title compound, which was recrystallized from ethanol solution as colourless plates (m.p. 528 K).

### 3. Refinement

All H atoms were positioned geometrically, with N—H = 0.86 Å, and C—H = 0.93 Å for aromatic H and refined using a riding model with *U*_iso_(H) = 1.2*U*_eq_(C, N) for aromatic and amide H.

### Crystal data

**Table:**

C_8_H_6_ClN_3_O	*Z* = 2
*M**_r_* = 195.61	*F*(000) = 200
Triclinic, *P*1	*D*_x_ = 1.571 Mg m^−^^3^
Hall symbol: -P 1	Melting point: 528 K
*a* = 6.5791 (4) Å	Mo *K*α radiation, λ = 0.71073 Å
*b* = 7.2663 (4) Å	Cell parameters from 1438 reflections
*c* = 9.3342 (5) Å	θ = 2.2–25.0°
α = 80.121 (4)°	µ = 0.42 mm^−^^1^
β = 85.042 (4)°	*T* = 296 K
γ = 70.235 (4)°	Plate, colourless
*V* = 413.52 (4) Å^3^	0.24 × 0.20 × 0.12 mm

### Data collection

**Table:**

Bruker SMART CCD diffractometer	1438 independent reflections
Radiation source: fine-focus sealed tube	1270 reflections with *I* > 2σ(*I*)
Graphite monochromator	*R*_int_ = 0.025
ω and φ scans	θ_max_ = 25.0°, θ_min_ = 2.2°
Absorption correction: ψ scan (*SADABS*; Sheldrick, 2007)	*h* = −7→7
*T*_min_ = 0.770, *T*_max_ = 1.000	*k* = −8→8
5938 measured reflections	*l* = −11→11

### Refinement

**Table:**

Refinement on *F*^2^	Primary atom site location: structure-invariant direct methods
Least-squares matrix: full	Secondary atom site location: difference Fourier map
*R*[*F*^2^ > 2σ(*F*^2^)] = 0.034	Hydrogen site location: inferred from neighbouring sites
*wR*(*F*^2^) = 0.094	H-atom parameters constrained
*S* = 1.06	*w* = 1/[σ^2^(*F*_o_^2^) + (0.0473*P*)^2^ + 0.1038*P*] where *P* = (*F*_o_^2^ + 2*F*_c_^2^)/3
1438 reflections	(Δ/σ)_max_ = 0.001
118 parameters	Δρ_max_ = 0.17 e Å^−^^3^
0 restraints	Δρ_min_ = −0.15 e Å^−^^3^

### Special details

**Table:**

Experimental. IR (KBr): 1686 (C=O), 3433 (NH); ^1^H-NMR (400 MHz, DMSO-D_6_, δ p.p.m.): 7.46–7.50 (d, 2H, ArH, J = 16 Hz), 7.90–7.94 (d, 2H, ArH, J = 16 Hz), 8.12 (s, 1H, C5H), 12.00 (s, 1H, NH); ^13^C-NMR (100 MHz, DMSO-D_6_, δ p.p.m.): 119.26, 128.77, 128.84, 136.66, 136.72, 152.17; MS (m/*z*, 70 eV): 197 (*M*^2+^), 195 (*M*^+^), 127, 125, 113, 111.
Geometry. All s.u.'s (except the s.u. in the dihedral angle between two l.s. planes) are estimated using the full covariance matrix. The cell s.u.'s are taken into account individually in the estimation of s.u.'s in distances, angles and torsion angles; correlations between s.u.'s in cell parameters are only used when they are defined by crystal symmetry. An approximate (isotropic) treatment of cell s.u.'s is used for estimating s.u.'s involving l.s. planes.
Refinement. Refinement of *F*^2^ against ALL reflections. The weighted *R*-factor *wR* and goodness of fit *S* are based on *F*^2^, conventional *R*-factors *R* are based on *F*, with *F* set to zero for negative *F*^2^. The threshold expression of *F*^2^ > 2σ(*F*^2^) is used only for calculating *R*-factors(gt) *etc*. and is not relevant to the choice of reflections for refinement. *R*-factors based on *F*^2^ are statistically about twice as large as those based on *F*, and *R*- factors based on ALL data will be even larger.

### Fractional atomic coordinates and isotropic or equivalent isotropic displacement parameters (Å^2^)

**Table:**

	*x*	*y*	*z*	*U*_iso_*/*U*_eq_	
Cl1	−0.31296 (10)	0.62029 (8)	1.39673 (5)	0.0705 (2)	
O2	−0.1420 (2)	0.9297 (2)	0.66886 (13)	0.0595 (4)	
N3	0.1322 (2)	0.79679 (19)	0.83947 (14)	0.0385 (3)	
N4	0.3528 (2)	0.7607 (2)	0.83651 (15)	0.0488 (4)	
N5	0.2213 (2)	0.8940 (2)	0.62018 (15)	0.0457 (4)	
H5	0.2196	0.9413	0.5291	0.055*	
C11	−0.1807 (3)	0.6731 (2)	1.23269 (18)	0.0462 (4)	
C10	−0.2983 (3)	0.7604 (3)	1.1128 (2)	0.0600 (5)	
H10	−0.4479	0.7927	1.1186	0.072*	
C9	−0.1960 (3)	0.8017 (3)	0.9812 (2)	0.0557 (5)	
H9	−0.2767	0.8615	0.8986	0.067*	
C8	0.0241 (2)	0.7544 (2)	0.97287 (16)	0.0369 (3)	
C7	0.0477 (3)	0.8791 (2)	0.70550 (17)	0.0414 (4)	
C12	0.0388 (3)	0.6240 (3)	1.2260 (2)	0.0600 (5)	
H12	0.1183	0.5645	1.3092	0.072*	
C13	0.1426 (3)	0.6632 (3)	1.0949 (2)	0.0563 (5)	
H13	0.2924	0.6278	1.0894	0.068*	
C6	0.3967 (3)	0.8216 (3)	0.70340 (19)	0.0500 (4)	
H6	0.5350	0.8162	0.6684	0.060*	

### Atomic displacement parameters (Å^2^)

**Table:**

	*U*^11^	*U*^22^	*U*^33^	*U*^12^	*U*^13^	*U*^23^
Cl1	0.0853 (4)	0.0835 (4)	0.0407 (3)	−0.0336 (3)	0.0192 (2)	−0.0029 (2)
O2	0.0439 (7)	0.0937 (10)	0.0378 (6)	−0.0286 (7)	−0.0083 (5)	0.0143 (6)
N3	0.0342 (7)	0.0481 (7)	0.0320 (7)	−0.0137 (5)	−0.0030 (5)	−0.0012 (5)
N4	0.0351 (8)	0.0675 (9)	0.0393 (8)	−0.0140 (6)	−0.0019 (6)	−0.0020 (6)
N5	0.0429 (8)	0.0615 (8)	0.0319 (7)	−0.0210 (6)	0.0008 (6)	0.0018 (6)
C11	0.0586 (11)	0.0470 (9)	0.0333 (8)	−0.0202 (8)	0.0072 (7)	−0.0046 (7)
C10	0.0396 (9)	0.0893 (14)	0.0466 (10)	−0.0202 (9)	0.0037 (8)	−0.0030 (9)
C9	0.0403 (9)	0.0861 (13)	0.0344 (9)	−0.0181 (9)	−0.0046 (7)	0.0041 (8)
C8	0.0399 (8)	0.0389 (8)	0.0313 (8)	−0.0132 (6)	−0.0013 (6)	−0.0029 (6)
C7	0.0411 (9)	0.0499 (9)	0.0337 (8)	−0.0187 (7)	−0.0030 (7)	0.0011 (6)
C12	0.0579 (12)	0.0775 (13)	0.0346 (9)	−0.0167 (10)	−0.0070 (8)	0.0096 (8)
C13	0.0399 (9)	0.0786 (12)	0.0412 (10)	−0.0149 (8)	−0.0072 (8)	0.0087 (8)
C6	0.0377 (9)	0.0696 (11)	0.0405 (9)	−0.0180 (8)	0.0025 (7)	−0.0041 (8)

### Geometric parameters (Å, º)

**Table:**

Cl1—C11	1.7444 (16)	C11—C12	1.364 (3)
O2—C7	1.237 (2)	C10—C9	1.385 (3)
N3—C7	1.367 (2)	C10—H10	0.9300
N3—N4	1.3833 (19)	C9—C8	1.369 (2)
N3—C8	1.419 (2)	C9—H9	0.9300
N4—C6	1.288 (2)	C8—C13	1.375 (2)
N5—C6	1.348 (2)	C12—C13	1.383 (3)
N5—C7	1.359 (2)	C12—H12	0.9300
N5—H5	0.8600	C13—H13	0.9300
C11—C10	1.352 (3)	C6—H6	0.9300
			
C7—N3—N4	111.34 (13)	C9—C8—C13	119.67 (15)
C7—N3—C8	128.99 (13)	C9—C8—N3	120.90 (14)
N4—N3—C8	119.63 (12)	C13—C8—N3	119.43 (15)
C6—N4—N3	103.86 (13)	O2—C7—N5	127.42 (15)
C6—N5—C7	107.98 (14)	O2—C7—N3	128.69 (15)
C6—N5—H5	126.0	N5—C7—N3	103.89 (13)
C7—N5—H5	126.0	C11—C12—C13	119.63 (17)
C10—C11—C12	120.83 (16)	C11—C12—H12	120.2
C10—C11—Cl1	119.12 (14)	C13—C12—H12	120.2
C12—C11—Cl1	120.04 (14)	C8—C13—C12	119.95 (17)
C11—C10—C9	119.95 (17)	C8—C13—H13	120.0
C11—C10—H10	120.0	C12—C13—H13	120.0
C9—C10—H10	120.0	N4—C6—N5	112.93 (15)
C8—C9—C10	119.94 (17)	N4—C6—H6	123.5
C8—C9—H9	120.0	N5—C6—H6	123.5
C10—C9—H9	120.0		
			
C7—N3—N4—C6	0.28 (18)	C6—N5—C7—N3	0.31 (18)
C8—N3—N4—C6	−177.50 (14)	N4—N3—C7—O2	179.93 (18)
C12—C11—C10—C9	−0.3 (3)	C8—N3—C7—O2	−2.6 (3)
Cl1—C11—C10—C9	−179.35 (16)	N4—N3—C7—N5	−0.37 (18)
C11—C10—C9—C8	−0.1 (3)	C8—N3—C7—N5	177.14 (14)
C10—C9—C8—C13	1.1 (3)	C10—C11—C12—C13	−0.3 (3)
C10—C9—C8—N3	−179.50 (17)	Cl1—C11—C12—C13	178.82 (16)
C7—N3—C8—C9	−2.1 (3)	C9—C8—C13—C12	−1.6 (3)
N4—N3—C8—C9	175.26 (15)	N3—C8—C13—C12	178.97 (17)
C7—N3—C8—C13	177.37 (16)	C11—C12—C13—C8	1.2 (3)
N4—N3—C8—C13	−5.3 (2)	N3—N4—C6—N5	−0.1 (2)
C6—N5—C7—O2	−179.98 (18)	C7—N5—C6—N4	−0.2 (2)

### Hydrogen-bond geometry (Å, º)

**Table:**

*D*—H···*A*	*D*—H	H···*A*	*D*···*A*	*D*—H···*A*
N5—H5···O2^i^	0.86	1.95	2.7924 (18)	166
C6—H6···O2^ii^	0.93	2.53	3.360 (3)	149
C9—H9···O2	0.93	2.29	2.933 (2)	126

Symmetry codes: (i) −*x*, −*y*+2, −*z*+1; (ii) *x*+1, *y*, *z*.
